# The Current Dilemma and Breakthrough of Stem Cell Therapy in Ischemic Heart Disease

**DOI:** 10.3389/fcell.2021.636136

**Published:** 2021-04-22

**Authors:** Chuanbin Liu, Dong Han, Ping Liang, Yang Li, Feng Cao

**Affiliations:** ^1^Medical School of Chinese PLA, Beijing, China; ^2^The Second Medical Center, Chinese PLA General Hospital, National Clinical Research Center for Geriatric Disease, Beijing, China; ^3^Department of Interventional Ultrasond, The Fifth Medical Center, Chinese PLA General Hospital, Beijing, China; ^4^Department of Cardiology, The Sixth Medical Center, Chinese PLA General Hospital, Beijing, China

**Keywords:** stem cell, ischemic heart disease, cardiac repair, cellular therapy, mechanism

## Abstract

Ischemic heart disease (IHD) is the leading cause of mortality worldwide. Stem cell transplantation has become a promising approach for the treatment of IHD in recent decades. It is generally recognized that preclinical cell-based therapy is effective and have yielded encouraging results, which involves preventing or reducing myocardial cell death, inhibiting scar formation, promoting angiogenesis, and improving cardiac function. However, clinical studies have not yet achieved a desired outcome, even multiple clinical studies showing paradoxical results. Besides, many fundamental puzzles remain to be resolved, for example, what is the optimal delivery timing and approach? Additionally, limited cell engraftment and survival, challenging cell fate monitoring, and not fully understood functional mechanisms are defined hurdles to clinical translation. Here we review some of the current dilemmas in stem cell-based therapy for IHD, along with our efforts and opinions on these key issues.

## Introduction

Ischemic heart disease (IHD) is the leading cause of death worldwide (Moran et al., 2013), and it is estimated to have 11 million patients with IHD in China (The Writing Committee of the Report on Cardiovascular Health and Diseases in China, 2020). Myocardial ischemia causes irreversible myocardial cell death, which leads to mass loss of heart function, formation of fibrous scars, and adverse cardiac remodeling, eventually progress toward heart failure (Tao et al., 2015; Han et al., 2017). Although many advances have been made in the medical and surgical treatment for IHD, some IHD patients are not suitable for surgery and have no effective drug treatment, they are called “no-option” patients (Fuchs et al., 2003). Stem cell therapy has, emerged as a new strategy for the treatment of IHD (Nguyen et al., 2016; Dou et al., 2018). However, clinical studies have not yet yielded the expected benefits, and multiple preclinical and clinical studies have shown paradoxical result (Pan et al., 2014; Bolli and Ghafghazi, 2017; Bagno et al., 2018). A meta-analysis by Gyöngyösi et al. (2016) assessed the efficacy of stem cells from different sources for the treatment of acute myocardial infarction (AMI). The results argued that stem cell therapy had no significant effects on the improvement of myocardial contractility, ventricular remodeling and clinical prognosis. Several clinical trials found that stem cell transplantation had a moderate or subtle improvement in the heart function of patients with AMI and chronic heart failure after 1 year’s follow-up (Cong et al., 2015; Henry et al., 2017). The ejection fraction of patients in the treatment group increased by 4–6% compared with the control group, and stem cell transplantation inhibited the early ventricular remodeling (Gyöngyösi et al., 2015). What do these discrepancies come from? The following unsolved issues of stem cell therapy might be able to answer this question.

## Cell Sources and Dosage

**FIGURE 1:**
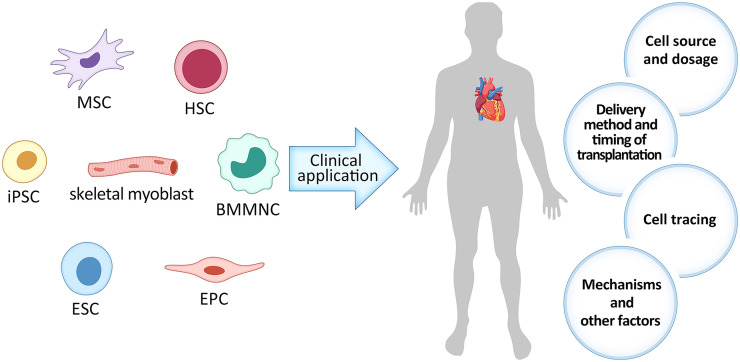
The current dilemma with stem cell therapy in ischemic heart disease (IHD). MSC, Mesenchymal stem cell; iPSC, induced pluripotent stem cell; ESC, embryonic stem cell; EPC, endothelial progenitor cell; HSC, hematopoietic stem cell; BMMNC, bone marrow-derived mononuclear cell.

Plenty of studies have shown that various cell types exerted beneficial effects on cardiac repair (Figure 1), such as skeletal myoblast (Suzuki et al., 2000), bone marrow-derived mononuclear cell (BMMNC) (Cao et al., 2009), hematopoietic stem cell (HSC) (Garikapati et al., 2013), endothelial progenitor cell (EPC) (Babin-Ebell et al., 2010), mesenchymal stem cell (MSC) (Shabbir et al., 2009), embryonic stem cell (ESC) (Menasché et al., 2015), induced pluripotent stem cell (iPSC) (Mauritz et al., 2011), etc. Skeletal myoblast was the first cell type to be clinically tested in myocardial repair since researchers surmised that it can increase myocardial contractility. However, the efficacy was unsatisfactory mainly due to the high incidence of arrhythmias (Suzuki et al., 2000). BMMNC contains the undifferentiated HSC and MSC as well as other committed cells in various stages of maturation. Its abundance and easy accessibility allows for autologous implantation without expansion, which avoids the decline of stem cell differentiation and migration ability and negated the incidence of immune rejection. In our previous clinical trial, we found that intracoronary delivery of autologous BMMNC was safe and feasible for STEMI patients and can lead to long-term improvement in myocardial function as far as 4 years (Cao et al., 2009; Tao et al., 2015). HSC has multiple differentiation potentials and can be autologously transplanted, but they are limited in abundance, which leads to poor efficacy (Garikapati et al., 2013). EPC, isolated from peripheral blood and bone marrow, can give rise to vascular cells. Clinical application of EPC transplantation is expected to increase the capillary density and subsequently improve the microcirculation around the transplanted sites in infarcted heart. Studies have showed that EPC transplantation can also improve heart function, but its effect is restricted, which may result from its weak differentiation ability (Babin-Ebell et al., 2010). ESC have strong proliferation and differentiation capabilities, but it has ethical controversies and high risks of teratoma formation, which create hurdles to its clinical translation (Hentze et al., 2007; Wang et al., 2013).

MSC can be isolated from multiple tissues including, but not limited to, bone marrow, adipose tissue and umbilical cord, and can be easily expanded *in vitro*. MSC is the most widely studied cell type for its abundant source, simple operation, and immune exemption (Shabbir et al., 2009). Plenty of studies have confirmed its effectiveness and safety. Leistner et al. (2011) used autologous BM-MSCs from 55 patients with AMI for vascular reconstruction after *in vitro* expansion and culture. Within 1 week, the cells were transplanted by means of intracoronary arterial injection. After 5 years of follow-up, no patient was hospitalized for treatment due to heart failure, and no cardiac muscle calcification or tumor formation was found. The ejection fraction of the patients increased from (46 ± 10)% to (57 ± 10)%, and the infarct area decreased significantly. 116 patients with AMI received the treatment of umbilical cord mesenchymal stem cells *via* coronary artery injection for 4 months, the results showed that the myocardial survival area and myocardial perfusion in the treatment group were better than those in the control group. After 18 months, the cardiac function test showed that the ejection fraction in the treatment group increased by 7.8%, while that in the control group only increased by 2.8%(Gao et al., 2015). Another important cell type for cell therapy is iPSC. It can be derived from patients themselves and resembles the characteristics of ESC. A large number of preclinical studies have confirmed that iPSC-derived cardiomyocytes can improve cardiac function after AMI (Khatiwala and Cai, 2016). Besides, it is easy to obtain and can be expanded in a large amount in a short time *in vitro*, which stands out as the most promising cell population in the future.

As far as we are concerned, the selection of seed cells must follow the disease-driven principles firstly. For example, HSC is preferred for leukemia, MSC is preferred for graft-vs.-host disease (GVHD), etc. For IHD, MSC, and iPSC have a good prospect. Secondly, the ideal seed cells of IHD need to meet the following requirements: high availability (“off-the-shelf”), not functionally impaired by patient’s comorbid conditions, consistent, convenient, and inexpensive large-scale expansion. According to the results of a number of stem cell clinical trials in the last decade, MSC and iPSC are the most promising cell types for IHD in clinical settings (Labusca et al., 2018).

Cell dose may be an important determinant of the efficacy of cell therapy. Excessive cells not only increase the costs, but also increase various risks, such as inducing vascular blockage, while too few cells may fail to achieve satisfactory results. However, there are few studies on the dose-effect relationship of cell transplantation and there is a lack of comparability between different studies due to differences in cell types, cell culture differences, transplantation methods, follow-up time, and evaluation indicators. A meta-analysis by Xu et al. (2016) involved the use of different dose of bone marrow stem cells (BMCs) for the treatment of AMI. The results showed that the LVEF change in the BMCs group was significantly higher than in the control group when the dose of BMCs was 1 × 10^8^ and 1 × 10^9^, while no significant LVEF change was observed at a dose of 1 × 10^7^. In addition, when the dose of BMCs exceeds 1 × 10^9^, they did not manifest significant LVEF up-regulation (*P* = 0.10). Therefore, stem cells may be effective in patients with AMI with a dose between 1 × 10^8^ and 1 × 10^9^. For the treatment of chronic IHD, Afzal et al. (2015) found that there was no significant improvement in cardiac parameters in trials that used fewer than 5 × 10^8^ cells. Transplantation of 5 × 10^8^ to 1 × 10^9^ cells induced significant improvement in LVEF and left ventricular end systolic volume (LVESV). In addition, different transplantation methods have posed different demands on cell dosage, and the optimal dosage remains to be further determined in each specific setting.

## Route of Cell Delivery and Timing of Cell Transplantation

**FIGURE 2:**
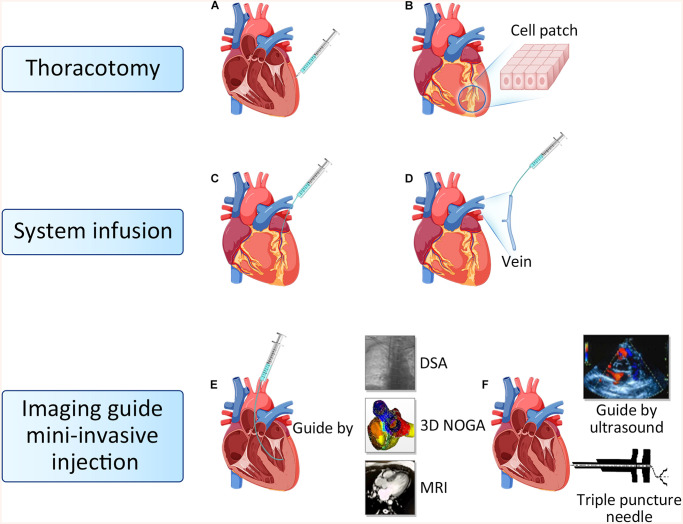
Route of cell delivery. **(A)** Epicardial intramyocardial injection. **(B)** Biological tissue engineering and cell patch. **(C)** Intracoronary injection. **(D)** Intravenous injection. **(E)** Endocardial intramyocardial injection guide by DSA, 3D NOGA and MRI. **(F)** Ultrasound guided targeted epicardial injection with a triple puncture needle device. DSA, Digital subtraction angiography; MRI, Magnetic resonance imaging.

To date, delivery routes in cardiac cell therapy mainly includes thoracotomy injection, system infusion and imaging guide mini-invasive injection (Figure 2; Kanelidis et al., 2017).

### Thoracotomy Injection

Thoracotomy injection consists of epicardial intramyocardial injection and cell patch. The trans-epicardial intramyocardial injection is the most classical cell delivery method, which can inject cells into the targeted area directly to avoid cells loss (Hamano et al., 2001). However, this method usually requires general anesthesia and thoracotomy. Potential adverse effects are left ventricular perforation, bleeding from the myocardium and unbalanced ventricular motion caused by the uneven distribution of cells after injection. This transplantation method is suitable for patients undergoing coronary artery bypass surgery and simultaneous heart valve surgery. Tissue engineering technologies have been used to develop cell delivery methods for regenerative therapy (Avolio et al., 2015). Using this approach, stem cells are expanded and adhered to cardiac patch, and subsequently delivered onto the surface of the damaged heart *via* thoracotomy. In our previous research, we designed a cardiac patch fabricated with electrospinning cellulose nanofibers modified with chitosan/silk fibroin (CS/SF) multilayers, then engineered with adipose tissue-derived mesenchymal stem cells (AD-MSCs). We adhered the nano-patch to the epicardium of the infarcted region in rat hearts, found that CS/SF-modified nanofibrous patches promote the functional survival of engrafted AD-MSCs (Chen et al., 2018). It has been reported that the cell patch improved cell survival and engraftment, resulting in a positive effect on cardiac function (Shimizu et al., 2009; Noguchi et al., 2016; Sugiura et al., 2016).

### System Infusion

System infusion including intracoronary injection and intravenous injection. Intracoronary injection can increase the number of cells homing to the ischemic area of the myocardium, while avoiding the damage caused by direct injection in the myocardium (Diederichsen et al., 2008; de Jong et al., 2014). Patients do not need to open the chest when combined with PCI surgery, so it is the most commonly used approach in clinical practices. The disadvantage is that some stem cells can be lost through coronary circulation, and over dose of cell injection can cause coronary artery occlusion, resulting in regional myocardial infarction again (de Jong et al., 2014). Intravenous stem cell transplantation is a non-invasive, reproducible, economical and convenient clinical treatment strategy for patients with IHD. Although some studies have shown that systemic intravenous administration of cells could hardly home to the heart because of the pulmonary first-pass effect (Lin et al., 2015). However, other studies argued that intravenous delivery of MSC could limit infarct size and improve LV function, which could be ascribed to the secreted TSG6 from trapped cells in lungs. TSG6 was subsequently proved to promote the revascularization of damaged heart (Lee et al., 2009). In our previous study, we combined intracoronary delivery with multiple intravenous infusions in a procine model of chronic myocardial ischemia, which was associated with improved cardiac function, increased perfusion, and alleviated ventricular remodeling, thus demonstrating the efficiency of combined delivery methods (Liu et al., 2016). Both intracoronary delivery and intravenous infusion are convenient in clinical practice, but whether the combination of both is more superior than one alone and whether multiple transplantation is better than single transplantation still need further examination.

### Imaging-Guided Mini-Invasive Injection

Imaging-guided mini-invasive injection includes trans-endocardial intramyocardial and trans-epicardial intramyocardial injection. Trans−endocardial intramyocardial injection guided by DSA, MRI, or three-dimensional (3D) NOGA electromechanical mapping system does not require thoracotomy, and stem cells can be transplanted directly into the target area, while it requires special positioning equipment and devices (Freyman et al., 2006; van der Spoel et al., 2012). During operation, there are potential risks such as myocardial cell damage, arrhythmia, and cell death of transplanted cells caused by high-pressure technology (Gyöngyösi et al., 2008). We have recently tested imaging-guided mini-invasive injection, and invented a triple puncture needle device that can perform intramyocardial injections through the thoracic epicardium under ultrasound guidance. With this device, only 2 or 3 holes are punctured in the chest wall of the subject each time, and cells can be injected into the myocardium through the thoracic epicardium under the guidance of ultrasound, and the injection position can be accurately controlled. It has the advantages of less trauma, fewer complications, and multiple transplantation at different time points. The optimal transplantation method requires non or mini invasive, high cell retention. Imaging guide mini-invasive injection is hope to be widely used in clinical practices.

The optimal timing of cell therapy after AMI has been investigated in several trials using BM-MSC with catheter-based applications. Myocardial microenvironment at different time points after infarction has profound influences on stem cells survival, homing and differentiation (Traverse et al., 2012; Khodayari et al., 2019). In acute infarct stage, the microenvironment is not conducive to the survival and growth of stem cells because of the overwhelming inflammatory response in the myocardial injury area. It was found that inflammatory reaction peaks at 1–4 days, some cytokines (such as VEGF) which were favorable to stem cells migration reached the peak of secretion at 7 days, and scars began to form at about 14 days after AMI (Traverse et al., 2009; Miettinen et al., 2012). In a recent systematic review, cardiac parameters [LVEF, LVESV, and left ventricular end-diastolic volume (LVEDV)] were significantly improved when stem cells were transplanted between 7 and 10 days after AMI (Traverse et al., 2011). For chronic IHD, there is no obvious time window problem, so we can select the time when the patients are in good condition (such as no angina attack and general physical activity without discomfort, which denotes that the heart blood supply and heart function are still good), suggesting that the patients’ internal environment and myocardial microenvironment are relatively favorable for transplantation, so as to facilitate the survival, homing and differentiation of implanted cells.

### Cell Tracing

**FIGURE 3:**
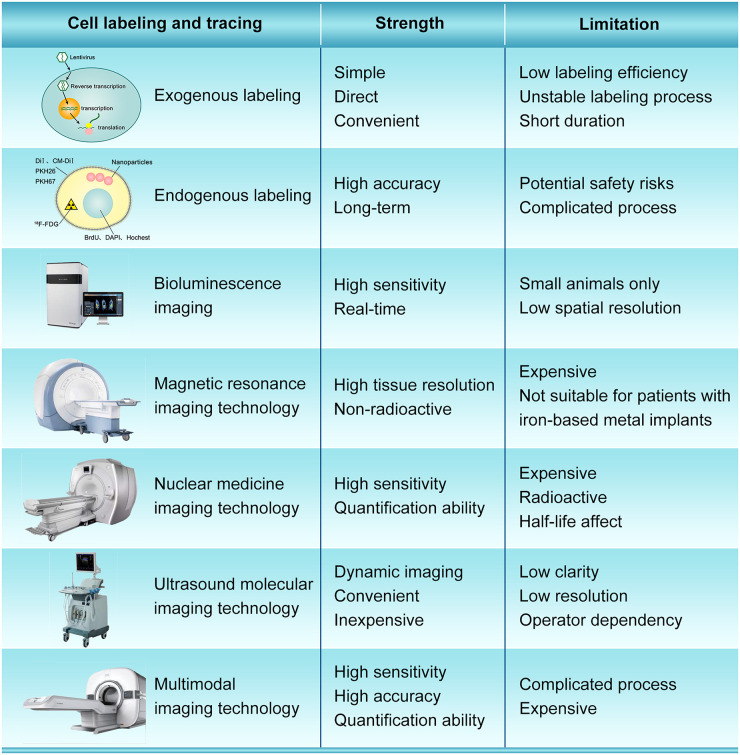
The strength and limitation of different cell labeling and imaging modality.

The cell fate after transplantation is still unclear, which can be partially ascribed to the deficiency of optimal cell labeling and tracing methods. The ideal stem cell labeling method should have the characteristics of simplicity, strong specificity, high sensitivity, neglectable toxicity, good anti-interference performance and low false positive rate. Currently, cell labeling strategies can be mainly divided into exogenous labeling and endogenous labeling (Figure 3). Exogenous cell labeling transfer the molecular probe labels into cells for direct labeling, such as fluorescent dyes, ^18^F-FDG radionuclide labels, nanoparticles, microbubble ultrasound contrast agents, etc. (Kircher et al., 2011; Qin et al., 2019). Fluorescein mainly bind to lipid molecules on the cell membrane, and the commonly used fluoresceins are DiI, CM-DiI, PKH26, PKH67 (Jiang et al., 2008; Weir et al., 2008). Fluorescent dye labeling is suitable for short-term tracing, because fluorescence will dilute and quench with the proliferation and differentiation of stem cells and the extension of time. The nucleic acid labeling method uses BrdU, DAPI, or Hoechst to label the DNA in the nucleus (Wang et al., 2016). With the increase of labeling time, nucleic acid labeling can also contaminate the surrounding cells. Besides, the labeled markers on transplanted cells may reduce or lost, which is very difficult to determine the stem cells that really need to be detected and observed. Radionuclide labeling has high resolution and accuracy, but its labeling time is limited by the short half-life, and radioactive elements may cause certain damage to stem cells and the human body (Hess et al., 2019). The method of labeling stem cells with nanoparticles is simple and can observe the migration of stem cells in the body non-invasively. However, there are some disadvantages. For example, the division of stem cells will dilute the concentration of nanoparticles in the cells, which will affect the long-term observation effect, and cell death will cause the dispersion of nanoparticles (Gallina et al., 2015; Zhu et al., 2016). In general, the exogenous labeling method is simple and direct, but it has disadvantages such as low labeling efficiency, unstable labeling process, short duration of the label in the cell, and difficulty in tracking the whole process.

Endogenous labeling use viral or non-viral vectors to transduce and integrate protein-encoding reporter genes into the cell genome, and mark cells through the overexpression of specific reporter genes, such as GFP fluorescent protein (Hayashi and Hamachi, 2012; Tao et al., 2015). The signal of the reporter gene does not disappear with cell division and proliferation, and the tracking accuracy is high, which can achieve long-term cell tracking and overcome some deficiencies of exogenous labeling. However, reporter gene imaging requires genome manipulation, which has potential safety issues and deserves attention (Cao et al., 2006; Youn and Chung, 2013). Besides, the Y chromosome is a specific genetic structure of male individuals, which is stable and persistent. Labeling the specific gene sequence on the Y chromosome to prepare probes can trace the labeled stem cells for a long time without special treatment for the stem cells (Lu et al., 2010). However, the stability and sensitivity of the Y chromosome probe need further experimental research and verification.

In recent years, advances in imaging equipment have allowed monitoring of stem homing and survival. *In vivo* imaging technology is widely used for tracing after stem cell transplantation, which includes bioluminescence imaging (BLI), magnetic resonance tracing technology, nuclear medicine tracing technology, computer tomography tracing technology, ultrasonic tracing technology, and multimodal tracing technology (Figure 3).

BLI uses visible light produced by luciferase to produce visible light at a specific wavelength for visualization, which has high sensitivity, and can track and quantify cell survival. It also has a rapid response to environmental changes, fast imaging speed, and clear images. BLI also has advantages in *in vivo* imaging, which can obtain real-time information of complex pathophysiological processes of living tissue non-invasively (Zhang et al., 2017). Iwano et al. (2018) using a red-shifted and highly deliverable luciferin analog performed directed evolution on firefly luciferase and designed an all-engineered bioluminescence *in vivo* imaging system (named AkaBLI), which produces *in vivo* emission signals 100 to 1,000-fold brighter as compared with conventional technology. The AkaBLI allowing non-invasive visualization of single cells deep inside freely moving animals, and recorded video-rate bioluminescent signals from neurons in the striatum, a deep brain area, for more than a year. However, this technology is only used for animal study, not yet been applied to clinics.

Magnetic resonance tracer cell imaging technology uses MR contrast agent to label cells, Mn^2+^, gadolinium chelate-based contrast agents, superparamagnetic iron oxide nanoparticles (SPIO), etc. MRI *in vivo* tracking of transplanted cells is a convenient, safe, non-invasive and sensitive method, which also has the advantages of high speed and high tissue resolution (Kim et al., 2007; Wallat et al., 2017). It can accurately locate and quantitatively analyze the lesions, and is non-radioactive. Skelton et al. (2015) found that the localization and dispersion of ferumoxytol-labeled cells could be effectively imaged and tracked at days 0 and 40 by MRI *in vivo*. However, MRI still has its own limitations: it cannot be dynamic in real time and not suitable for patients with any type of iron-based metal implants.

Nuclear medicine imaging technology refers to the use of radionuclide as tracers, which is suitable for functional and metabolic imaging. The most widely used radionuclide tracers include ^99m^Tc, ^18^F-FDG, ^125^I, etc., but these radionuclide tracers have certain radioactive contamination and toxicity (Lezaic et al., 2013; Skelton et al., 2015; deKemp et al., 2016). The technology need two devices, single electron emission computed tomography (SPECT) and positron emission tomography (PET), both of which have high sensitivity (Katsikis and Koutelou, 2014).

Ultrasound molecular imaging technology connects specific ligands to the surface of ultrasound contrast agents smaller than red blood cells, accumulates in target tissues through blood circulation to observes its specific imaging (Lee et al., 2014). The key to this technology is ultrasound contrast agents, of which nano-scale ultrasound contrast agents are widely used. Ultrasound imaging technology can trace monitor stem cells in real time, and is a non-invasive, non-toxic, non-radioactive, dynamic imaging method with high sensitivity. However, the image quality of ultrasonic tracing technology is inferior to other technologies in terms of clarity and resolution, and is greatly affected by gas, as well as the technical level of the operator (Li Y. et al., 2018; Brown and Lindner, 2019).

The above-mentioned molecular imaging technologies have their unique advantages for stem cell tracking. However, when one of these imaging technologies is used alone, its inherent shortcomings cannot be ignored for imaging. Therefore, some scholars have proposed a multimodal imaging mode, which refers to the fusion of two or more imaging modes. The advantages of these combined modes are complementary, which can solve the shortcomings of a single imaging mode and improve the sensitivity and resolution of tissue imaging. Multimodal imaging technology has great advantages and potential for tracking stem cells *in vivo*. At present, PET/CT and SPECT/CT have been widely used in clinic. The key to multimodal imaging is multifunctional contrast agents. In recent years, due to the rapid development of multifunctional contrast agents, many scholars have studied ultrasound (US)/MRI (Mahmoudi et al., 2015), US/MRI/fluorescence (Sung et al., 2009), US/CT/MRI (Tang et al., 2014), and other multimodal stem cell tracing modes (Tao et al., 2016; Li et al., 2017; Qiao et al., 2017; Su et al., 2017). For pre-clinical research, the more detail the better, while for clinical applications, the more convenient the better. Therefore, in our opinion, multimodal molecular imaging will be the predominant tool for tracing and evaluating efficacy after cell transplantation.

## Mechanism of Stem Cell Therapy

**FIGURE 4:**
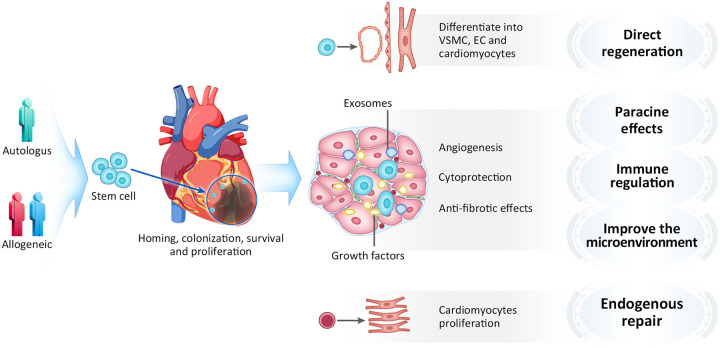
Mechanism of stem cell therapy. VSMC, Vascular smooth muscle cell; EC, Endothelial cell.

The mechanism of cell-based myocardial repair is very complicated, and the underlying details remained to be uncovered. Attention to transplanted cells has shifted from the capacity of *in situ* differentiation toward cardiomyocytes and other vascular components, to their capacity of secreting factors that affects surrounding myocardium in a paracrine manner. The primary mechanisms by which cell therapy exerts beneficial effects is now believed to include direct regeneration, paracrine effects, immune regulation, microenvironment improvement and promoting endogenous cardiac repair (Ni et al., 2017; Müller et al., 2018; Figure 4). Most of implanted cells were lost due to washing out from injected area and cell death from ischemia and inflammation. It is thought that very few numbers of stem cells might be able to directly contribute to vascular structures, and there are no clinical evidence that transplanted stem/progenitor cells differentiate into new mature myocardial so far. To date, paracrine effects is considered as important functional mechanism of cellular therapy (Gnecchi et al., 2008; Farzaneh et al., 2019). The transplanted stem cells released cytokines, chemokines and growth factors that inhibit apoptosis and fibrosis, enhance contractility (Han et al., 2017). Studies have revealed that stem cells can secrete a variety of cytokines, such as VEGF, epidermal growth factor (EGF), and hepatocyte growth factor (HGF) (Tang et al., 2015; Shafei et al., 2017), which play important roles in stem cell proliferation, chemotaxis, differentiation and anti-apoptosis. Besides, stem cells can also secrete anti-inflammatory factors, such as IL-10, which can regulate myocardial inflammation after myocardial infarction and improve the microenvironment (Bishopric, 2008; Li S. et al., 2018). Additionally, stem cells can secrete exosomes, which play important roles in cytoprotection, stimulation of angiogenesis, induction of antifibrotic cardiac fibroblasts, and modulation of M1/M2 polarization of macrophages infiltrating the infarcted region (Yu et al., 2014; Khan et al., 2015; Rosca et al., 2015; Zhao et al., 2015; Suzuki et al., 2016; Barile et al., 2017; Derkus et al., 2017; Jung et al., 2017; Menasché, 2018). Exosomes are membrane-bound extracellular vesicles with a diameter of 30–100 nm, contain biologically active proteins, RNAs and microRNAs, which involved in cell-to-cell communication. Studies have shown that exosomes secreted by mesenchymal stem cells can protecting myocardial cells from apoptosis and promoting cell proliferation and angiogenesis. Recently, Gao et al. (2020) found that using the exosomes naturally produced from that mixture of heart muscle cells, endothelial cells and smooth muscle cells which were all derived from hiPSCs yields regenerative benefits equivalent to the injected hiPSC-CCs in a porcine model of myocardial infarction. Vagnozzi et al. (2019) recently found that transplanting live stem cells, dead stem cells, and zymosan (a chemical inducer of the innate immune response) can all provided a similar functional rejuvenation to the heart after ischemia-reperfusion injury in mice. Interestingly, injection of stem cells followed by immunosuppressant cyclosporine A eliminated this beneficial effect, which indicates that the immune regulation may be an important mechanism. Their study demonstrated that the functional benefit of cardiac cell therapy is due to an acute inflammatory-based wound healing response that rejuvenates the mechanical properties of the infarcted area of the heart. In addition, the heart itself has stem cells, which can slowly self-renew (Rafatian and Davis, 2018). A radioactive isotope study demonstrated that human cardiomyocytes gradually renew 1% of the cell population annually at the age of 25 years to 0.45% at the age of 75 years, which suggests that the heart has the limited ability of endogenous repair (Bergmann et al., 2009). However, some studies found that the stem cell transplantation could activate and promote endogenous myocardial repair (Wang et al., 2014; Wu et al., 2018; Sebastião et al., 2019). In our view, the limited self-renewal ability of the heart is obviously insufficient to support the improvement of short-term function, we believe that its repair mechanism may have a close association with paracrine effects and immune regulation.

## Future Clinical Perspectives

As the population ages, the number of IHD patients is increasing. Stem cell therapy, as a promising treatment method, will receive more and more attention. MSCs, especially umbilical cord mesenchymal stem cells (UC-MSCs), have the characteristics of multiple differentiation capabilities, immune exemption, easy access and large-scale expansion, and ethical requirements. In the future, they are expected to become a new drug for the treatment of IHD. iPSCs derived from patients themselves, are easy to obtain and do not have immune rejection. They can differentiate into a variety of cells and even organoids under the induction of small molecule compounds. They have been widely used in drug screening and efficacy evaluation. Generation of thick vascularized tissues that fully match the patient still remains an unmet challenge in cardiac tissue engineering. By means of 3D bioprinting, Noor et al. (2019) successfully print a cellularized human heart with major blood vessels using cardiac and endothelial cell-laden hydrogels, which demonstrate the potential of the approach for engineering personalized tissues and organs. Besides, mechanical and biochemical stimulation on stem cell improve the efficiency of tissue engineering, which provides new strategy for the current stem cell therapy dilemma (Rinoldi et al., 2019). In the future, stem cells can combined with tissue engineering and 3D printing technology, which will have broad application prospects in tissue regeneration and organ transplantation. In addition, the exosomes provide an acellular therapeutic option for myocardial injury since they are acellular, consequently, easy to store and transport, which also avoiding the tumorigenic risks, arrhythmogenic complications, and immune rejection. With the development of imaging, minimally invasive and even non-invasive cell transplantation guided by imaging will be the trend. At the same time, multimodal imaging for cell tracing also provides convenient conditions for elucidating the mechanism of stem cell action.

## The Risks of Stem Cell Therapy

As for the stem cell therapy, we should not only consider effectiveness and clinical accessible, but also pay more attention to the safety, including the risks of teratoma formation, arrhythmogenic complications, immune rejection, etc. The tumorigenesis post stem cells transplantation is one of the major concern to the clinical translation application, especially for ESC and iPSC. In order to reduce the risk of teratoma formation, there are three main strategies currently. Firstly, induce terminal differentiation or completely remove the residual pluripotent cells before transplantation. Secondly, interfere with the tumorigenic genes in the residual pluripotent stem cells (Hagan et al., 2018). Thirdly, do a good job of monitoring the formation of tumors after cell therapy, and clear them once they are found. For example, introducing suicide genes into stem cells, once tumors are formed, specific drugs can be used to safely remove tumors and prevent spreading. In addition, Tao et al. (2018) found that compared with ESC, parthenogenetic embryonic stem cell (pESC) have similar differentiating capacity but meanwhile with the risk reduction of teratoma formation and without ethical controversy, which provide a new choice for cell sources. Arrhythmogenic complications of cell therapy was firstly reported in skeletal myoblast (Suzuki et al., 2000). Majority studies have shown that MSC transplantation for IHD does not increase the risk of arrhythmia. Ramireddy et al. (2017) found that there was no evidence for ventricular proarrhythmia, manifested by sustained or non-sustained ventricular ectopy or worsened heart rate variability (HRV) in patients receiving MSCs. However, the long-term effects of cell therapy on ventricular arrhythmia need to be further studied. In addition, stem cell transplantation combined with amiodarone and ivabradine to prevent arrhythmia is also a feasible strategy.

## Conclusion

Although stem cell therapy in IHD has achieved positive results and showed good prospects for clinical application, the optimum cell source and cell dosage, route of administration and timing of transplantation, cell tracing and the mechanism are parameters that still need to be addressed. Besides, we should also pay attention to the cell-preparation techniques, patients selection, follow up observation time, functional evaluation methods, and safety. Furthermore, disappointing results from clinical trials thus far suggest that we should perhaps reconsider fundamental strategies to overcome the hurdles while continuing to expand the application of stem cell therapy for future clinical trials.

## Author Contributions

This work was collaboration among all of the authors. CL wrote the initial draft of the manuscript and prepared the figures. DH, PL, and YL reviewed the manuscript. FC proposed the original idea and reviewed the manuscript. All authors read and approved the final submitted version of the manuscript.

## Conflict of Interest

The authors declare that the research was conducted in the absence of any commercial or financial relationships that could be construed as a potential conflict of interest.
